# Reconfigurable Microfluidic Magnetic Valve Arrays: Towards a Radiotherapy-Compatible Spheroid Culture Platform for the Combinatorial Screening of Cancer Therapies

**DOI:** 10.3390/s17102271

**Published:** 2017-10-04

**Authors:** Alexandre R. Brunet, Frédérique Labelle, Philip Wong, Thomas Gervais

**Affiliations:** 1 Biomedical Engineering Institute, École Polytechnique de Montréal, Montreal, QC H3T 1J4, Canada; alexandre.r-brunet@polymtl.ca; 2 Institut du Cancer de Montréal, Université de Montréal, Montréal, QC H3T 1J4, Canada; pwong.kf@gmail.com; 3 Centre de Recherche du Centre Hospitalier de l’Université de Montréal, Montréal, QC H2X 0A9, Canada; 4 Department of Engineering Physics, École Polytechnique de Montréal, Montréal, QC H3T 1J4, Canada; frederique.labelle@polymtl.ca; 5 Department of Radiation Oncology, Centre Hospitalier de l’Université de Montréal, Montréal, QC H2X 0A9, Canada

**Keywords:** microfluidics, radiotherapy, chemotherapy, spheroids, drug discovery, valves

## Abstract

We introduce here a microfluidic cell culture platform or spheroid culture chamber array (SCCA) that can synthesize, culture, and enable fluorescence imaging of 3D cell aggregates (typically spheroids) directly on-chip while specifying the flow of reagents in each chamber via the use of an array of passive magnetic valves. The SCCA valves demonstrated sufficient resistance to burst (above 100 mBar), including after receiving radiotherapy (RT) doses of up to 8 Gy combined with standard 37 °C incubation for up to 7 days, enabling the simultaneous synthesis of multiple spheroids from different cell lines on the same array. Our results suggest that SCCA would be an asset in drug discovery processes, seeking to identify combinatorial treatments.

## 1. Introduction

Cancer treatment has evolved with an increasing number of molecular targeted agents being approved for different diseases [1]. Molecular targeted agents are often developed as monotherapies first, then explored in combination with standard cytotoxic chemotherapies (CT), but seldom with radiotherapy (RT). Nevertheless, there have been examples where combinations of drug/drug [2] or drug/RT [3,4,5,6] during a treatment led to a synergistic effect to either reduce drug toxicity [3] and/or induce a stronger response against cancerous cells than when treatments were used independently. Such results show that some drugs, whose development was halted because a lack of efficacy as monotherapy, could still hold the potential to treat cancer when combined with other drugs or RT. Furthermore, it is well established that cancer cell in monolayer culture tend to be significantly more sensitive to RT than typical in vivo tissue [7,8,9,10]. Therefore, tridimensional (3D) models, such as spheroids, are expected to better assess treatment efficacy as they present more cell-to-cell interactions [8,9,10,11] and could thus hold the key to improved drug screening.

In addition, in the context of microfluidics, spheroids of various sizes can easily be obtained in large numbers [10,12,13] at relatively low costs. In devices made exclusively of polydimethylsiloxane (PDMS), the natural permeability of the material to gas (O_2_ and CO_2_) permits culture medium replenishment every 24 h or more instead of requiring a constant perfusion [13,14].

**Figure 1:**
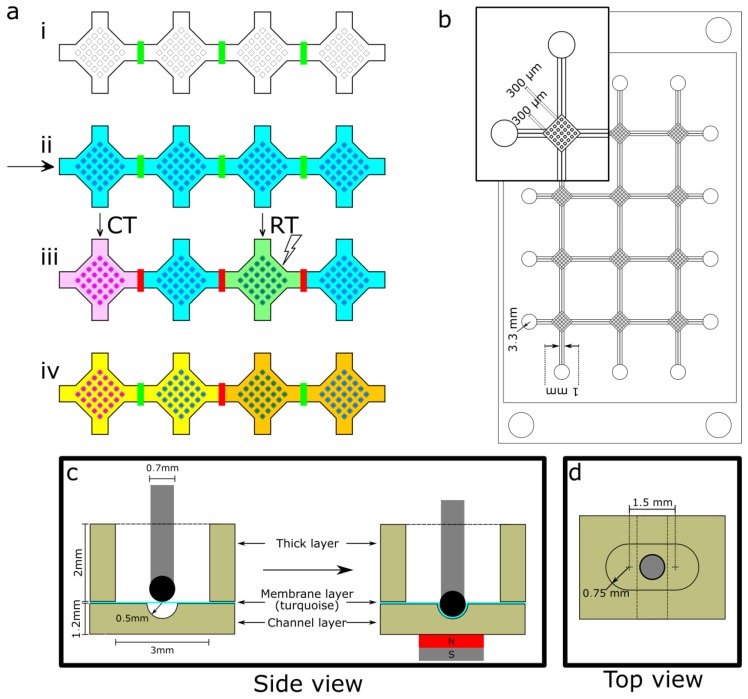
Elements involved in a spheroid culture chamber array (SCCA) design. (**a**) Working principle of a microfluidic SCCA: (i) all valves are open (shown in green between each culture chamber containing 5 × 5 wells) to fill the device with a cell suspension; (ii) cells are left to sediment in each well to form spheroids; (iii) some valves are closed (depicted in red), so that different treatments (chemotherapy (CT) or radiotherapy (RT)) can be applied to different columns of culture chambers independently; (iv) each valve can be opened independently to create a specific flow path for a reagent through the device; (**b**) Mold used for SCCA’s channel layer; (**c**) Side view of the microfluidic valves used: when a magnet is inserted under the channel, the magnetic polydimethylsiloxane (mPDMS)-tipped (black) metal rod gets pulled down, deforming the membrane and closing the channel; (**d**) Top view of microfluidic valves used, the ovaloid opening represents the hole through which the metal rod (grey) is inserted.

In order to study treatment synergy in a high throughput format, spheroid forming microfluidic chips must also enable combinatorial testing of drug interactions. Microfluidics enable the use of valves that can separate culture chambers, creating an M × N array A for which each element $a_{mn}$ corresponds to a different culture condition [15,16]. An example of how CT and RT can be performed on a spheroid culture chamber array (SCCA) is shown in Figure 1a.

Microfluidic valves can be divided into actively and passively actuated, the former requiring an external power source to be actuated. Active valves can generally be remotely controlled and can be actuated via pressure [15,16,17,18], heat [18,19], or magnetism using solenoids [18,20,21]. Many active valves have already been integrated into arrays and multiplexers for monolayer cultures [15,16]. However, since they need external power sources to be actuated, they are more expensive and it can be challenging to have them operated by non-specialists. On the other hand, passive microfluidic valves do not need external power sources to be operated, making them easier to handle. They are typically actuated mechanically, chemically or magnetically. Mechanic valves are typically closed by a user that presses on or screws into the actuator [19]. Chemically actuated passive valves respond to a change in concentration of an effector (typically pH) in the surrounding fluid [22]. Both types are slower to operate than magnetic valves, which are usually more compact, resist to higher pressures and are easily toggled between opened/closed states [23,24,25].

In this study, we designed and fabricated a microfluidic SCCA entirely of PDMS (Figure 1b) combined with a passive magnetic valve array that enables 3D cell cultures. Each culture chamber can grow up to 25 spheroids under the same condition. We demonstrate a simple valve design that enables easy manipulation and reversible separation of culture chambers within the device at low cost, which could lead to study of up to 12 treatment conditions on the same SCCA.

To ensure reliability of the valves, their resistance to pressure was tested under various conditions relevant to cell culture (different incubation times and RT doses). The diffusion of small particles across the valves was also studied in order to ensure complete separation of samples during a study. Finally, flow routing and spheroid culture experiments were performed in the device to demonstrate its potential.

## 2. Materials and Methods

### 2.1. Description of the Design

#### 2.1.1. Valve Design

The designed passive magnetic valve was inspired by Gaspar et al. [25] and uses a permanent magnet and a metal rod for actuation, as shown in the side view (Figure 1c). The actuation of the valve is triggered by inserting the metal rod into an opening on top of the channel (Figure 1d) and a magnet underneath. The attraction between the two deforms a membrane, closing the valve. This design was selected for its low cost and complexity level, compactness and high resistance to pressure. Valves are also individually addressable to increase the versatility of the system. The valve consists of a first layer of 1.2 mm of PDMS (channel layer) that contains a semi-cylindrical microchannel (radius of 0.5 mm) of at least 2 mm, topped with a 100 μm-thick PDMS membrane (turquoise line in Figure 1d) and another layer of 2 mm of PDMS (thick layer) that contains a 1.5 mm × 3 mm ovaloid opening. To create a conformal seal of the channel by the membrane, the tip of the metal rod was rounded by adding a drop of magnetic PDMS (mPDMS, see Section 2.2.4).

#### 2.1.2. Spheroid Culture Chamber Arrays (SCCA)

We developed a 5.06 × 7.62 cm^2^ (2 × 3 in^2^) SCCA that contained four vertical and three horizontal semi-cylindrical channels (valves), each interconnected and a few millimeters long (Figure 1b). At the end of each channel, there were 3.3 mm inlets and at the junction of each channel were diamond-shaped culture chambers. Cells were cultured into spheroids in each chamber, which consisted of 25 (5 × 5) cubic square wells of 300 µm on a side separated by 300 µm. The size of the wells was chosen as small as possible to match the resolution of the printer and to be able to keep the channel layer as thin as possible to maximize the magnetic force. Between each chamber was a valve to isolate culture media, resulting in a flexible flow control in the device. The chambers were separated in order to be compatible with the spatial resolution of RT devices and to facilitate manipulation of the valves.

### 2.2. Fabrication and Assembly

#### 2.2.1. Molds and Substrates

SCCAs were fabricated out of PDMS (Sylgard 184 Silicone Elastomer Kit, Dow Corning, Auburn, MI, USA). First, the molds were designed on a CAD software (Catia V5R20, Dassault Systèmes, Vélizy-Villacoublay, France) and the STL files were then processed using the Asiga software (Asiga Composer 1.2.3, Alexandria, Australia). A layer thickness of 50 µm was chosen and PLASClear v2.0 was selected as the resin. The parts were then 3D printed individually by using an Asiga Pro2 for the SCCA and an Asiga Freeform Pico 39 for the individual test valves (ITV) to evaluate resistance to burst. Both were post-processed as recommended by Comina et al. [26]. Briefly, the device was dehydrated in two consecutive 5 min baths in isopropanol, followed by a 5 min curing step under UV light, a 5 min incubation in a heated 70% ethanol ultrasonic bath, a quick dry using a nitrogen gun and an application of a thin layer of paint (Aztek opaque black, Testors, Vernon Hills, IL, USA) using a generic dual-action gravity feed airbrush. This post-processing was necessary in order to harden the molds and allow PDMS to cure properly during the molding step.

#### 2.2.2. Device Molding and Assembly

**Figure 2:**
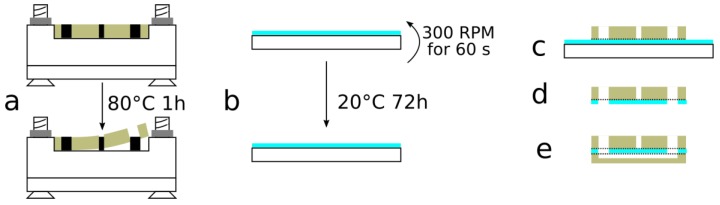
Fabrication and assembly of the spheroid culture chamber array (SCCA): (**a**) The 3D printed molds of the thick and channel layers were screwed onto polymethylmetacrylate (PMMA) plates, polydimethylsiloxane (PDMS) at 10:1 was poured inside the molds, cured at 80 °C for 1 h and removed from the molds; (**b**) For the membrane layer, PDMS at 20:1 was poured over a silanized glass wafer before being rotated at 300 rpm for 1 min and cured at room temperature for 72 h; (**c**) The thick layer and the membrane layer (still mounted on its substrate) were plasma bonded together; (**d**) Holes corresponding to the inlets/outlets were punched into the membrane layer using the thick layer as a guide; (**e**) The other side of the membrane layer and the channel layer were plasma bonded together.

The thick layer and channel layer were constructed by pouring PDMS mixed at a 10:1 ratio into the 3D printed molds and allowing it to cure at 80 °C for 1 h (Figure 2a). For the SCCA, the molds were screwed to a polymethylmetacrylate (PMMA) plate to prevent the PLASClear from curling up when heated and minimize variation in the thickness of the layers. The substrate used for membrane fabrication was a 4-inch glass wafer that was silanized for 24 h by using trichloro(octadecyl)silane under vacuum. PDMS mixed at a 20:1 ratio was poured over the silanized glass wafer to create the membrane layer (Figure 2b). Afterwards, the wafer was spin-coated using a Cee 200CBX (Brewer Science, Rolla, MO, USA) at 300 rpm for 60 s and was cured at room temperature for 72 h.

#### 2.2.3. Device Assembly

Both the thick layer and the channel layer were removed from their respective molds using tweezers. The layers were bonded by following the sequences showed in Figure 2c–e and by using an atmospheric plasma (Dyne-A-Mite, Enercon, Menomonee Falls, MI, USA). The surfaces to be bonded were first exposed to the plasma for 60 s for SCCA and 30 s for the ITV and then pressed together. First, the thick layer was bonded on the membrane (still attached to its substrate; Figure 2c), then the assembly was detached from the substrate using a scalpel, and holes were punched into the membrane at the spots corresponding to the inlets/outlets (Figure 2d). Afterwards, the membrane/thick layer were manually aligned and bonded to the channel layer using the atmospheric plasma (Figure 2e).

#### 2.2.4. Valves Reusable Parts

The metal rods were created from sections of paper clips tipped with drops of mPDMS. First, a section of 2 cm of the paper clip was cut using metal scissors, producing a metal rod with a flat and a sharp end. The flat end of the metal rod was dipped in uncured mPDMS, obtained by mixing 10:1 PDMS with magnetite (Fe_3_O_4_, Alpha Chemicals, Cape Girardeau, MO, USA) at a 50/50 weight ratio [20,27]. The metal rod was then put in an oven at 80 °C for 1 h.

The magnets used for this project were 6.35 mm (1/4 in) square neodynium magnets (B444-N52, K&J Magnetics, Pipersville, PA, USA) and chosen because of their small size and high pull force. In order to place the magnets directly under the corresponding valves, a magnet-holder of PMMA was designed. The file for the magnet-holder was generated in Inkscape (Version 0.91, Inkscape: Open Source Scalable Vector Graphics Editor) before being uploaded to a Trotec Speedy 300 CO_2_ laser cutter (Trotec, Marchtrenk, Austria), which was used to cut the part from a 6.35 mm PMMA plate. Once the plate was made, the magnets were inserted manually to ensure that each magnet’s polarity would be the opposite of the adjacent magnets. The metal rods, neodymium magnets and magnet holder were all reusable and could be sterilized using conventional methods, even though this was not tested in the present study, since these parts were never in direct contact with fluids or biologic material.

### 2.3. Experimental Procedure

#### 2.3.1. Experimental Protocol for Valve Resistance

**Figure 3:**
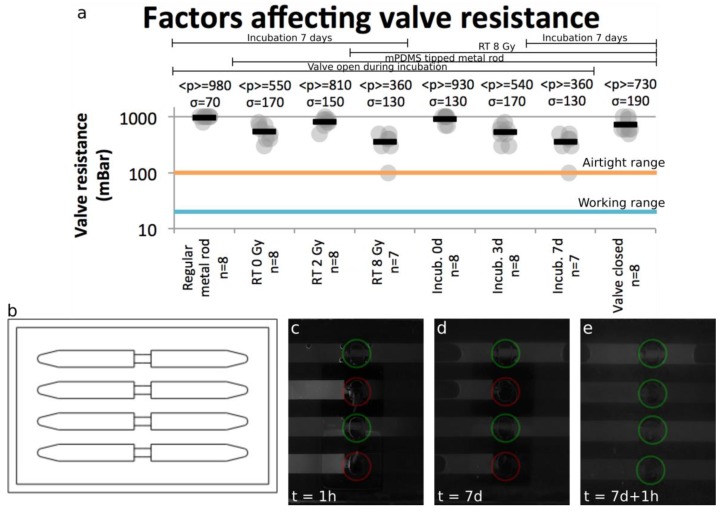
Characterization of valve resistance to flow and diffusive mass transfer (all data shown) (**a**) Effect of various parameters (type of metal rod used, radiotherapy (RT) dose, incubation time and state of valve in the incubator) over valve resistance in mBar (1 mBar = 1 cm of H_2_O); (**b**) Schematics of device channel layer mold used for individual test valves (ITV); (**c**–**e**) Small molecule (333 Da) diffusion across the closed valve. The valves in green represent open valves, while the ones in red are closed.

The ITVs (Figure 3b) were used to evaluate the valves’ resistance to applied pressures and diffusion of small molecules. Through the course of the experiments, the procedure described in Table 1 was followed, with the valves being left open and the devices left untouched during incubation, unless otherwise specified.

RT was performed using a Gammacell 3000 (Best Theratronics, Kanata, ON, Canada). Valve resistance was tested on day 7 for each device by using an AF1 dual unit pressure pump (Elveflow, Paris, France) and visualized in bright field using a stereoscope (Olympus SZX16, Olympus, Tokyo, Japan). To observe valve resistance, the pressure pump was connected to one end of the channel and liquid was allowed to fill the channel until it crossed the valve. The pump was stopped while closing the valve and the applied pressure increased until a movement of the meniscus was perceptible under the microscope. Every 20 s, the applied pressure was increased by increments of 10 from 0 to 100 mBar and by increments of 100 from 100 to 1000 mBar, which corresponds to the maximum pressure attainable with the pump that was used.

To observe diffusion through the valve, immediately after RT treatment, half of the valves (two out of four) in each device were closed and a drop of a fluorescein solution (M422-05, JT Baker, Center Valley, PA, USA) was deposited into each inlet (left of the device). The systems were then imaged in green fluorescence using the aforementioned stereoscope, and put into the incubator at 37 °C, 5% CO_2_. After 7 days, the devices were removed from the incubator and imaged with the same stereoscope. The valves were then opened and the systems were imaged one hour later.

#### 2.3.2. Experimental Protocol for SCCA

For the SCCA validation, experiments were divided into two categories: one for actual cell culture and one for flow control (using food dyes). The SCCA was prepared by following the same procedure as the ITVs (Table 1), with the addition of plastic connectors at each inlet/outlet. For the flow control experiments, the valves of the device were adjusted in different configurations and filled with food dye solutions. During the experiments, the plastic connectors were left in the inlets (top and right), but were removed from the outlets (bottom and left) to induce a variation of pressure, which facilitated the flow of liquid rather than flow by diffusion, which is time consuming. During the whole experiment, the outlets were frequently emptied using a pipette in order to maintain fluid flow.

For the cell culture experiments, after RT treatment, the SCCA was seeded with untreated cell suspensions of the ovarian cell lines OV1946 [28] at 2.5 × 10^6^ cells/mL and OV90 [29] at 5 × 10^6^ cells/mL. These cell lines were genetically engineered to express enhanced green fluorescent protein (OV1946 and OV90) and mCardinal (OV90). The device was incubated at 37 °C, 5% CO_2_ for 24 h with all valves within horizontal channels closed. Subsequently, the medium was replaced and the devices were imaged using a Nikon ECLIPSE TE300 microscope (Nikon, Tokyo, Japan) in bright field and fluorescence using a Nikon super high pressure mercury lamp. Cell medium was replaced every 24 h, before imaging.

## 3. Results and Discussion

### 3.1. Valve Resistance

From the valve resistance results, we determined two limits that described valve behavior under pressure. The *working limit* is defined as a resistance of 20 mBar. We set this limit since a regular device should not exceed 10 cm in length and the pressure that would be generated by tilting the device at 90° should be of approximately 10 mBar (1 mBar being equivalent to 1 cm pressure head in H_2_O). By adding a security factor of two, we obtain the said limit of 20 mBar. The *airtight limit* represents a valve resistance of 100 mBar and was defined as the pressure that could be applied by softly pressing the surface of the system as to remove air bubbles. These limits provided restrictions and guidelines on device handling.

To validate the valve design and its function, we compiled all data from valve resistance (Figure 3). Mainly four factors can influence valve resistance: the metal rod used, the RT dose, the incubation time and the valve state (opened/closed) in the incubator. Each of these aspects was tested in ITVs made of four separate channels shut by a 2-mm valve placed at mid-point in each channel (Figure 3b). PDMS delamination at the edges of the main channel occurred at pressures nearing 500 mBar when the bonding between the PDMS layers failed before valve burst (around 5% of cases). In such cases, the valve resistance was defined as the highest pressure attained at a safe level before device rupture. In 50% of the results for pressures of 900 mBar and above, the valves appeared to resist the maximum pressure (1000 mBar) for a few seconds, but then leaked quickly. This could be attributed to a delamination of the PDMS around the valve. Although the valve itself would not directly cause this, valve resistance was again defined, using a worst case scenario approach, as the pressure level before the leak was detected (900 mBar).

#### 3.1.1. Pressure Resistance

As shown in the two first columns of Figure 3a, the use of a regular metal rod resulted in a higher valve resistance than the ones tipped with mPDMS. This was to be expected, since a higher iron density corresponds to a higher pull force. However, this increase in resistance was offset by a greater tendency for the valves to tear along the rod’s edges. The tearing was observed when the ITVs were exposed to longer incubation times or higher temperatures (data not shown). Since valve robustness and reusability is a more stringent design constraint than burst pressure, which is easily achieved experimentally (Figure 3a), soft, round mPDMS tipped rods offer a significant improvement in device performance.

Figure 3a demonstrates the relationship between valve resistance, RT dose and incubation time. As the dose increased from 2 to 8 Gy, the valve resistance decreased. This effect is in contradiction with what was observed by Briganti et al. [30], where a slight and non-significant difference between irradiated and untreated PDMS was observed We hypothesize that these changes could be attributable to an increase in polymer cross-linking in the bulk of the PDMS leading to an increase in brittleness, as observed elsewhere [31]. At RT doses of up to 8 Gy, the device was systematically capable of withstanding pressures over 100 mBar, which represented the airtight limit.

We also tested the effect of incubation length at 37 °C on the valve resistance. There appeared to be a direct correlation between time spent in the incubator and valve resistance. This relationship was expected as PDMS hardens over time and the curing process is greatly sped up when the temperature increases. Figure 3a also shows that the device resistance stayed over 100 mBar, the airtight limit, for a period of up to 7 days in the incubator, which was compatible with typical cell culture conditions. These results demonstrated that the valve resistance of our device achieved airtight limits even after being exposed to RT doses of 8 Gy and 7 days incubation at 37 °C.

The last aspect to consider when evaluating valve resistance was the state of the valve between uses. The two last columns of Figure 3a show that if a valve was kept in closed state during incubation, the resistance was greater than if kept in open state. When kept in closed state for a long period of time, the PDMS membrane took and held the shape of the channel, causing it to become more resistant in future operations. In order to ensure that the channels kept in closed position were still usable, we filled them with an aqueous solution and did not notice any impact on the flow when compared to the ones that were kept opened. This result showed that, whenever possible, valves should be kept in closed state during incubation, which is normally desired to ensure reagent segregation inside the chambers.

#### 3.1.2. Diffusion Resistance

Figure 3c–e shows the difference in diffusion-based transport of a fluorescein solution between opened and closed valves. A fluorescein solution that was loaded into the inlets (left) of each channel while the valves were left opened (first and third channels) or closed (second and fourth channels). As shown in Figure 3c, one hour after the injection, fluorescein had diffused across the whole channels when valves were kept open, but could not reach the far side of the closed ones. One week later (Figure 3d), the far side still remained untouched by fluorescein, but air (black meniscus) was present in the channel. After opening the valves, diffusion occurred in the whole channels (Figure 3e), showing that the channel could still be opened. The test conclusively demonstrated that the valves can prevent the diffusion of molecules of at least 333 Da (molecular mass of fluorescein) [32] and, consequently, could likely resist the diffusion of most biological signaling factors, such as cytokines (8–50 kDa), implicated in a variety of cellular pathways and signaling. This experiment also highlighted the occurrence of evaporation in the device, which could be explained by the breathability of PDMS. This side effect was compensated in cell culture experiments by changing the medium in the system every 24 h.

### 3.2. Fluid Routing Using Valve Arrays in the SCCA

**Figure 4:**
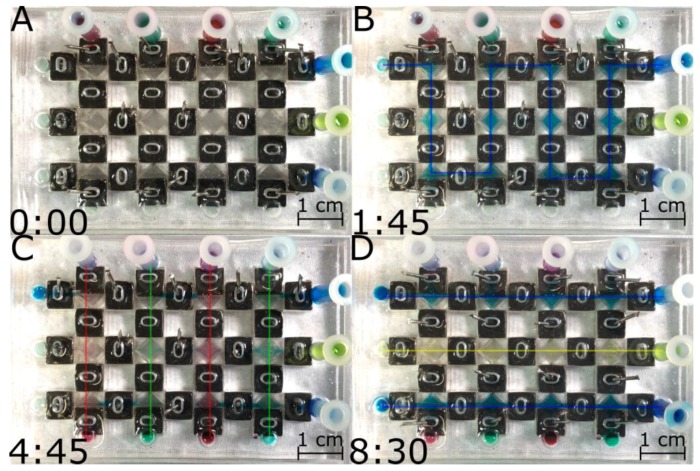
Screen captures from a video (see Supplementary Video S1) illustrating a typical flow rerouting experiment inside a SCCA (**A**) Device before loading at *t* = 0 min; (**B**) Loading of the device with blue dye through one inlet (top right) and one outlet (top left) at *t* = 1.75 min; (**C**) Vertical loading (top to bottom) of the device with red and green dyes at *t*= 4.75 min; (**D**) Horizontal loading (right to left) of the device with blue and yellow dyes at *t* = 8.5 min. The arrows indicate the direction of the flow in the channel and the color of the aqueous solution used.

After characterizing valve performance on ITVs, we proceeded to determine their collective behavior to reroute flows inside the SCCA. We applied a sequence of changes in the path conformation of channels of a SCCA to reroute aqueous solutions. Figure 4 shows the selection of flow paths realized during the course of a given experiment where four different reagents (illustrated by the dyes) were used to combinatorially generate four different reagent conditions in individual chambers (see Supplementary Video S1). It was possible to fill the entire device from one inlet (Figure 4b), the vertical inlets (Figure 3c) and the horizontal inlets (Figure 3d). No reagents leaked or followed a path other than the desired one. It would also be possible to do RT directly on chip with the use of a linear accelerator that can target individual compartments within the SCCA (not shown). In theory, up to M × N different reagents, cell lines, and RT doses combinations could be created using this matrix array.

**Figure 5:**
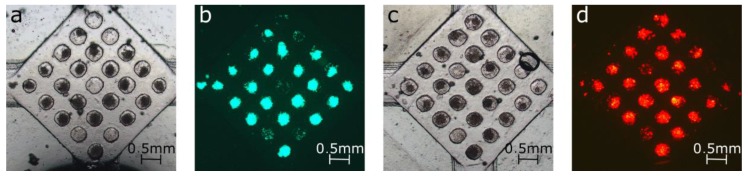
Cell culture in chamber array after 24 h incubation and after change of medium: OV1946 seeded at 2.5 × 10^6^ cells/mL in (**a**) bright field and (**b**) fluorescence; OV90 seeded at 5 × 10^6^ cells/mL in (**c**) bright field and (**d**) fluorescence.

The SCCA enabled the culture and bright light and fluorescent imaging of spheroids from different cell lines on one device. As illustrated in Figure 5, spheroids of OV1946 and OV90 were cultured and imaged directly on the same chip after 24 h, after change of cell medium. Both cell suspensions formed spheroids in the SCCA within 24 h. The cell density of the suspension injected in the system controlled the size of the spheroids and the dimensions of the well-defined maximum size attainable. Around 25% of the wells were empty by the time of the imaging, which could be caused by insufficient trapping, due to the size of the spheroids formed. In order to grow bigger spheroids, the size of the wells and the suspension density should be increased, since optimal trapping is achieved for spheroids whose diameter is at most half the size of the wells [14]. However, such a change affects the distance between the metal bar and the magnet and would require a slight increase in the magnetic field to maintain similar valve resistance and reliability.

## 4. Discussion and Conclusions

We have designed a microfluidic cell culture chamber array with passive magnetic valves that can be manipulated individually and resist pressures of at least 100 mBar after exposure to RT doses of 8 Gy and incubation at 37 °C for up to a week. We evaluated our device for the growth of spheroids and assessment of RT treatments directly on-chip. Our device was compatible with conditions that permit cell culture experiments for testing different treatment combinations simultaneously. It holds the potential to simultaneously study any M × N number of treatment conditions on the same chip, even though a 3 × 4 array was defined as the optimal configuration for a 5.06 × 7.62 cm^2^ footprint. The current bottleneck in chip fabrication is the membrane curing time (72 h), yet these dead times could be avoided in large scale manufacturing, as membranes could be pre-synthesized. Thus, the SCCA using permanent magnetic valve array is a promising platform for drug discovery or personalized medicine for identifying combinatorial cancer treatments on spheroid forming cells, which may find applications in other fields of microfluidics where magnetic valving needs to be combined with a see-through configuration for chip imaging.

## Figures and Tables

**Table 1:** Device preparation and experimental procedure.

Day 0	Assembly of the device Air bubbles removal through manual filling with 70% ethanol and isopropanol Injection of pluronic solution (10 mg/mL) Incubation for 24 h at room temperature
Day 1	Flush with water to remove excess pluronic Fill with cell medium (DMEM/F12) RT treatment of 0 to 8 Gy (8 Gy considered default)
Day 1–X	Incubation at 37 °C for 0 to 6 days (6 days considered default)

## Supplementary Materials

The following are available online at www.mdpi.com/1424-8220/17/10/2271/s1, Table S1—Valve resistance data, Video S1—Flow control experiment with aqueous dye solutions.
